# Cancer patients’ use of complementary and alternative medicine in Sweden: a cross-sectional study

**DOI:** 10.1186/s12906-019-2452-5

**Published:** 2019-03-13

**Authors:** Kathrin Wode, Roger Henriksson, Lena Sharp, Anna Stoltenberg, Johanna Hök Nordberg

**Affiliations:** 1 Regional Cancer Center Stockholm Gotland, Box 6909, 102 39 Stockholm, Sweden; 2 Department for Upper Gastrointestinal Cancer, K42, Karolinska University Hospital, 141 86 Stockholm, Sweden; 3 Department of Radiation Sciences, Umeå University, 901 87 Umeå, Sweden; 4 Department of Nursing, Umeå University, 901 87 Umeå, Sweden; 5 Department of Learning, Informatics, Management and Ethics, Division of Innovative Care Research, Karolinska Institutet, 171 77 Stockholm, Sweden; 6 Department of Neurobiology, Caring Sciences and Society, Division of Nursing, 23 300 141 83 Huddinge, Sweden; 7 Department of Physiology and Pharmacology, Karolinska Institutet, 171 77 Stockholm, Sweden

**Keywords:** Complementary and alternative medicine/utilization, Oncology, Cross-sectional studies, Cancer, Adult, Integrative oncology, Sweden, Europe, Epidemiology, Evidence-based medicine

## Abstract

**Background:**

Access to and advice on Complementary and Alternative Medicine (CAM) are uncommon within Swedish conventional cancer care and little is known about cancer patients’ own use of CAM. The aim of this cross-sectional study was to explore Swedish cancer patients´ patterns of CAM use, their experiences and preferences.

**Methods:**

Questionnaires were distributed consecutively to 1297 cancer patients at a university hospital’s out-patient oncology units. The response rate was 58% (*n* = 755). Descriptive statistics were used to analyze the survey data. A logistic regression model was used to investigate the association between CAM use and gender, age and level of education. Open-ended responses were analyzed, using qualitative content analysis.

**Results:**

Lifetime CAM use was reported by 34% (*n* = 256), and 26% (*n* = 198) used CAM after cancer diagnosis. Being female, younger and having higher education predicted CAM use. Most commonly used methods were natural products including vitamins and minerals and relaxation. Main reasons for CAM use were improvement of physical, general and emotional wellbeing and increasing the body’s ability to fight cancer. Satisfaction with CAM usage was generally high. Reported adverse effects were few and mild; 54% of users spent < 50 Euro a month on CAM. One third had discussed their CAM use with cancer care providers. More than half of all participants thought that cancer care providers should be able to discuss (58%) and to consider (54%) use of CAM modalities in cancer care.

**Conclusions:**

Despite limited access and advice within conventional cancer care, one fourth of Swedish cancer patients use CAM. The insufficient patient-provider dialogue diverges with most patients’ wish for professional guidance in their decisions and integration of CAM modalities in conventional cancer care. Concurrent and multimodal CAM use implies challenges and possibilities for cancer care that need to be considered.

**Electronic supplementary material:**

The online version of this article (10.1186/s12906-019-2452-5) contains supplementary material, which is available to authorized users.

## Background

Complementary and Alternative Medicine (CAM) is a broad set of non-mainstream practices including use of natural products, mind-body therapies and entire medical systems [1]. Use among cancer patients has increased in the last decades [2]. National and regional heterogeneity, gender, age, education and type of tumor appear to influence usage patterns and frequency [2–4]. Research on both effectiveness and risks of specific CAM modalities for cancer patients accumulates [5] and attempts to establish evidence-based clinical guidelines are made [6] and have recently been endorsed by the American Society of Clinical Oncology [7]. Open communication between patients and cancer care providers (beneath referred to as providers) has been valued essential to meet patients´ needs and to improve understanding regarding direct risks as well as to prevent indirect risks [8]. The authors of several studies [9–12] highlight the need of an improved dialogue concerning CAM. Leading comprehensive cancer centers [13] provide the concept of integrative oncology [14] as a patient-centered health care model to meet patients’ preferences, to ensure their safety and to optimize clinical outcomes [15–18]. Other authorities have established research centers and scientific information services about CAM [1, 19].

Previous studies on Swedish cancer patients’ CAM use have mainly focused on natural products [20] and specific groups of patients [10, 20] and indicate similar usage frequency as other high-income countries. Research on Swedish professionals´ perceptions of CAM indicate uncertainty about evidence, indications, contraindications and skepticism about high costs [21–23] .

Practice of evidence-based medicine requires integrating individual clinical expertise, patient values and circumstances with the best available external clinical evidence from systematic research [24]. To understand patient values and circumstances in relation to CAM it is crucial to study usage across different countries and cultures. This cross-sectional study builds on previous surveys to advance the view of cancer patients’ CAM use in general, focusing on Swedish cancer patients’ experiences and preferences.

## Methods

### Participants and inclusion

**Fig. 1:**
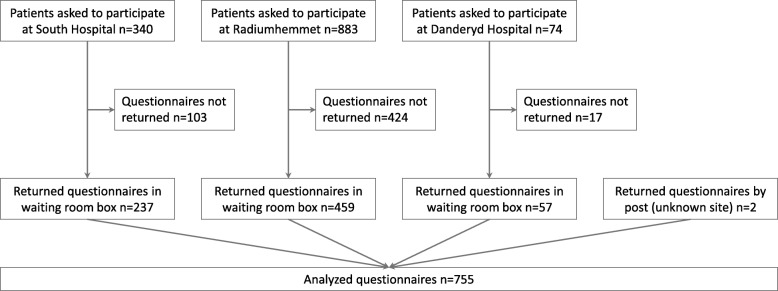
Flow chart of data inclusion

Data were collected from the three Oncological out-patient units (Fig. 1) at the Karolinska University Hospital, Stockholm with 14,614 patients during 2014 covering the region of Stockholm with 2.3 million of 10 million inhabitants in Sweden.

Inclusion criteria for study participants were re-visits to oncologist or nurse implying active oncological treatment (radiotherapy, chemotherapy and other medical cancer therapies) or follow-up; curative or palliative stage of the disease; solid tumor (breast cancer, gynecological - urogenital –, or gastrointestinal cancer, head-neck cancer, lung cancer, skin cancer, thyroid cancer, sarcoma, tumors with unknown primary or brain tumor). Exclusion criteria were first-time visits since we wanted to explore CAM use *after* cancer diagnosis, and treatment visits for chemotherapy and radiotherapy in order to avoid duplicate answers.

### Data collection

The study employed a cross-sectional design. Participants were asked to fill in a questionnaire with 19 questions including yes/no and multiple-choice responses as well as free text options (Table 1).

**Table 1:** Measures derived from data collection with questionnaire

Measures	Q No	Question	Response options	Additional free text option
Patient characteristics	1	Age	Open	
	2	Gender	Man/woman	
	3	Cancer site	Open	
	4	Highest level of education	Multiple choice	
CAM use	5	Use of complementary or alternative medicine at any point in life	Yes/No	
	7	Use of specific CAM therapy before and/or after cancer diagnosis, and/or in present time	Multiple choice^a^	X
	8	When was CAM initiated?	Open	
Adverse effects	11	Adverse effects	Yes/No^b^	X
Dialogue about CAM	15	Communication about CAM with conventional health care providers	Yes/No	
	15a	If communication, what was it about?	Open	
	15b	If communication, were you satisfied with the dialogue?	Yes/No	X
	15c	If not, why did you not communicate about CAM?	Open	
Sources of information	16	Source of information about CAM	Multiple choice	X
	18	Desired information pathway regarding CAM	Multiple choice	X
Reasons	9	Reason for CAM use	Multiple choice	X
	6	Main reason(s) for not using complementary and alternative medicine	Multiple choice	X
Benefits, satisfaction, costs	10	Perceived benefit of CAM therapy	Multiple choice^c^	X
	14	Satisfaction with CAM use after cancer diagnosis	Multiple choice	X
	12	Expenditure on CAM methods per month	Multiple choice	
	13	Perception whether CAM use was worth the money	Yes/No	X
Role of cancer care in relation to CAM	17	Conventional health care providers should be able to answer questions about CAM	Multiple choice	
	19	View on the role of conventional health care in relation to providing some CAM	Yes/No	X

^a^ List of 27 specific methods, space for additional therapies and specifications e.g. on type of herbal remedy or vitamin/mineral; ^b^ Free text option for specification of therapy and adverse effect; ^c^ Eight choices including no benefit at all

### Questionnaire content

The questionnaire previously used by Molassiotis et al. [3] was translated to Swedish and further adapted by the research team to suit the purpose of the present study and the local context. We excluded 8 items on background questions (income, ethnic group, religious beliefs and previous cancer treatment), 2 items regarding frequency of CAM use and 4 items concerning sick-leave, hospitalization and other health care visits. Moreover, 6 items on CAM use were merged into 1 item. The questionnaire ultimately contained 19 questions on demography, CAM use, reasons, used methods and details on experiences of and views on CAM (Table 1). An additional file shows the questionnaire in detail (Additional file 1).

### Data collection procedures

During one week (September 2014), all patients with appointments for cancer treatment or follow-up were informed verbally by the receptionist at all three out-patient clinics (written information about the study, voluntary participation and confidentiality). Patients willing to participate, completed the anonymous questionnaire in the waiting room and left it into a designated post box. There was no opportunity offered to complete the questionnaire at home.

### Statistics and data analysis

Data was summarized by descriptive statistics (frequencies and percentages). To investigate factors possibly associated with CAM use, Spearman correlation coefficients were calculated for CAM use versus gender, age and level of education and possible associations between these variables were explored with a logistic regression model. All calculations were done in STATA®.

Participants’ free-text responses were analyzed descriptively question-by-question according to principles of qualitative manifest content analysis by two researchers (KW and JHN) independently [25, 26]. The length of the free-text responses varied from one word to a few sentences. After reading all responses and compiling them into meaning units, the two researchers agreed upon a coding scheme for each response section. The codes within each section were then compared and contrasted and sorted into categories. The categories including example statements representing each category are presented in Table 2. These results were used for better understanding of quantitative data and as control whether there were missing options in the multiple-choice questions.

**Table 2:** Categorization of free-text responses with example statements

Question	Category	Examples of statements within category
Q9. Reasons for CAM use (n = 13, N = 198)	Specification of improved physical well-being (*n* = 12)	“To counteract strong hot flushes.”
	Refraining conventional treatment (n = 1)	“Did not want conventional treatment because I didn’t want any more poison in my body.”
Q10. Benefits of CAM (*n* = 31, N = 198)	Specification of physical and emotional well-being (*n* = 14)	“Less pain and better mobility.”
	Some CAM of value, some not (n = 2)	“Have experienced many side-effects from the antiestrogen treatment but my sexual life has not been affected, my mucous membranes are not dry. Because of the primrose oil? Not of any use: The acupuncture that even hurt sometimes.”
	I do not know (yet) (n = 15)	“Difficult to know what it would have been like without [CAM]. If it had any effect or not.”
Q13. Was CAM worth the money? (*n* = 54, N = 198)	CAM use significant in life (n = 15)	“I got a new life. Even if I would die tomorrow, it would have been worth it.”
	Improvement of psychological, physical or spiritual well-being (n = 24)	“Yoga makes me feel at peace and improves strength and flexibility and gives me a sense of having power.”
	Some CAM of value, some not (n = 2)	“The chiropractic practice helped me for some time with my wryneck, but the antioxidants were not worth the money.”
	Wish for economical support for CAM use (n = 6)	“My economy cannot take anti-cancer foods in the long run.”
	I do not know (yet) (n = 7)	“Too early to evaluate.”
Q14. Satisfaction with CAM use (n = 31, N = 198)	Specification of effect (n = 12)	“Did not notice the benefit before I stopped taking this mistletoe extract. Then I started again.”
	I don’t know (yet) (n = 14)	“Difficult to say as a lay person.”
	CAM use not in association to cancer (n = 5)	“I have not used CAM for cancer.”
Q15a. Topic of discussion with cancer care professionals (*n* = 49, *N* = 79)	Use of specific method was encouraged (n = 12)	“The doctor encouraged me to use acupuncture.”
	Ok to use (*n* = 10)	“The doctor thought it was totally ok.”
	Patient asked to take own responsibility for use (n = 10)	“Not his field but did not discourage me [from CAM use].”
	Recommendation to refrain usage (incl risk of interaction) (*n* = 17)	“The doctor said no to everything except what the Oncology department offered.”
Q15b. If discussion, were you satisfied? (*n* = 27, N = 79)	Specification of discussion about specific method (n = 6)	“Got an answer to my vitamin D in my blood. In the end, I stopped taking vitamin D since the test showed too much.”
	Lack of knowledge about CAM among health care providers (n = 7)	“Without nuance, uninformed and non-empathic. You don’t have to recommend complementary methods if you are so afraid of them before time has passed and additional 20 research results have proven benefits. But you could report about current research and where one can find research reports. I have been asking for this but have not gotten any help.”
	Wish for open attitude and competent answers (n = 14)	“There is research in the rest of the western world that is genuine. /…/ Swedish doctors/nurses would benefit from being open to alternatives.”
Q15c. Why not discussed? (*n* = 77, *N* = 119)	Expected negative answer (*n* = 18)	“Everyone knows about the lack of knowledge [about CAM] among doctors and their out-of-date attitudes regarding alternatives that do not constitute medicines or surgery. Unnecessary when one needs their support and not their irritation and skeptical attitude.”
	No reason to discuss (*n* = 44)	“The staff has the attitude: If you think it helps, then…”
	Nobody asked (*n* = 8)	“I have not gotten the question.”
	Lack of time or continuity (n = 7)	“Lack of time and lack of interest [from providers].”
Q16. Sources of information about CAM (n = 26, N = 198)	Own experience and interest (n = 15)	“Big interest in my whole life.”
	Literature, lectures, courses, patient organizations (n = 9)	“Books like: Anti-cancer, Are Waerland, Maesegården.”
	Other therapists (n = 2)	“My personal trainer.”
Q19. View on the role of conventional health care in relation to providing some CAM (*n* = 89, N = 198)	Important with evidence, competence and quality (n = 42)	“Good with holistic perspectives and sound scientific view on these methods. Otherwise one easily goes to quacks.”
	Suggestion of method and/or indication (*n* = 34)	“Important to offer all help that supports the fighting of cancer, especially considering all difficult hospital visits.”
	As provider of information on CAM (n = 10)	“Tell me what there is, and I can make the decision myself.”
	Wish for treatment diversity (n = 3)	“Right now, there is only one alternative. There needs to be options.”

n = number of free-text responses, N = number of responses to multiple choice/yes/no question

### Measures

Participants´ characteristics and CAM use were assessed by question (Q) 1–8, adverse effects by Q11, dialogue about CAM and sources of information by Q15, 16 and 18; reasons for CAM use, perceived benefits, satisfaction and monthly costs by Q9, 10, 12–14 and the role of cancer care in relation to CAM by Q17 and 19 (see Table 1).

## Results

### Participants´ characteristics and CAM use

Out of 1297 eligible patients, 58% (*n* = 755) returned the questionnaires. Content and response options are shown in Table 1, patient characteristics in Table 3.

**Table 3:** Patient characteristics

Patient characteristics	Patients % (n)	CAM user % (n)	No CAM user % (n)	Spearman’s correlation coefficient, rs	*p*-value
Age in years				−0.25^a^	< 0.01
< 30	2 (12)	2 (4)	1 (8)		
30–49	17 (130)	30 (59)	13 (71)		
50–69	46 (344)	46 (91)	45 (253)		
> 70	31 (234)	16 (32)	36 (202)		
Unknown	5 (35)	6 (12)	4 (23)		
Total	101 (755)	100 (198)	100 (557)		
Sex				0.22^b^	< 0.01
Women	64 (484)	80 (158)	59 (326)		
Men	34 (259)	17 (34)	40 (225)		
Unknown	2 (12)	3 (6)	1 (6)		
Total	100 (755)	100 (198)	100 (557)		
Diagnosis					
Breast cancer	38 (285)	51 (101)	33 (184)		
Urogenital cancer	18 (138)	8 (16)	22 (122)		
Gastrointestinal cancer	14 (103)	9 (18)	15 (85)		
Gynecological cancer	12 (92)	16 (31)	11 (61)		
Head, neck, lung or skin cancer	12 (92)	11 (21)	13 (71)		
Sarcoma	0 (2)	1 (1)	0 (1)		
Unknown	6 (43)	5 (10)	6 (33)		
Total	100 (755)	100 (198)	100 (557)		
Highest education				0.14^c^	< 0.01
Elementary school	18 (137)	8 (16)	22 (121)		
High school	31 (233)	31 (62)	31 (171)		
College/University	49 (368)	57 (112)	46 (256)		
Unknown	2 (17)	4 (8)	2 (9)		
Total	100 (755)	100 (198)	100 (557)		

^a^rs calculated using age as a continuous variable; ^b^rs calculated with a positive correlation for women; ^c^rs calculated using 4 categories with increased value for higher highest education

We found no statistically significant gender difference between eligible patients (65% women, 35% men) and participants (64% women, 34% men, 2% unknown).

Use of CAM over lifetime was reported by 34% (*n* = 256) of the participants and 26% (*n* = 198) had used CAM after their cancer diagnosis (beneath referred to as CAM users). Onset of CAM use was specified by 77/198 CAM users; the majority stated either a time correlation to cancer diagnosis or to cancer treatment. We found that 31 different CAM modalities were used by 198 patients after cancer diagnosis. Most frequently used CAM modalities were vitamins and minerals, natural products and relaxation (Table 4). Each modality may in itself represent many different variations, such as different types of mindfulness or yoga. “Vitamins and minerals” for example, implied usage of 22 different substances and the option “natural products” 32 different products.

**Table 4:** Distribution of used CAM modalities. Categories according to National Center for Complementary and Integrative Health [1]

Used CAM modalities	% (n)
Category Natural products	
Vitamins, minerals	10.1 (66)
Natural products	9.3 (61)
Injection of mistletoe preparations	1.8 (12)
Aromatherapy	0.8 (5)
Category Mind and Body Pratices	
Relaxation	9.0 (59)
Massage	8.7 (57)
Yoga	6.9 (45)
Meditation	6.7 (44)
Acupuncture	6.1 (40)
Mindfulness	5.3 (35)
Prayer	5.3 (35)
Naprapathy, chiropractic	4.0 (26)
Tai chi, Qigong	3.7 (24)
Spiritual guidance, healing	2.6 (17)
Supporting group(s)	1.7 (11)
Art therapy	1.4 (9)
Zone therapy	0.9 (6)
Rosen Method Bodywork	0.3 (2)
Shiatsu	0.3 (2)
Hypnosis	0.2 (1)
Feldenkrais method	0.2 (1)
Hyperthermia	0.2 (1)
Acupressure	0.2 (1)
Eurythmy therapy	0.2 (1)
Category Other Complementary Health Approaches	
Changes in diet	7.9 (52)
Anthroposophic medicine	2.4 (16)
Homeopathy	1.4 (9)
Energy medicine	0.8 (5)
Traditional Chinese Medicine	0.8 (5)
Ayurveda	0.6 (4)
Laser therapy	0.6 (4)
Total used modalities	100 (656)

We found statistically significant correlations between use of CAM and being female (rs = 0.22, *P* < 0.01), younger (rs = 0.25, P < 0.01) and having a higher education (rs = 0.14, P < 0.01). This was confirmed by logistic regression model with gender, age and level of education as predictors and usage of CAM as a dependent variable (*p* < 0.01). The model explains the variation of CAM users vs. No CAM users with 6%.

### Adverse effects

A total of 5.6% (*n* = 9) CAM users reported 11 adverse effects related to a CAM modality. Five reports related to gastrointestinal symptoms from mung bean sprout juice, iron, apricot pits, low-carb-high-fat diet during chemotherapy and one unknown remedy, respectively. Fever and shivering were reported from mistletoe, cough and morning fatigue from cannabis and pain from acupuncture needles. Two reports regarded undesirable effects of more reflective character, since detoxification from spirulina and possible toxification from intake of pesticides via fruits and vegetables was mentioned as adverse effects without reference to concrete symptoms. Finally, one report concerned an x-ray finding of a kidney stone and a reflection on overconsumption of spinach as possible cause.

### Dialogue about CAM and sources of information

Among CAM users, 33% (*n* = 66/198) had discussed CAM with their physician or nurse compared with 2% (*n* = 13/557) among No CAM users.

Responses regarding the providers’ (physician or nurse) reactions to CAM related questions, ranged from approval or advice that CAM use was one’s own responsibility to recommendation to refrain use. A general concern among participants was that they thought providers ought to be more open and knowledgeable about CAM (see Table 2 Q15b). One participant stated: “There is research in the rest of the western world that is genuine /…/ Swedish doctors/nurses would benefit from being open to alternatives.”. Participants´ main reasons for not discussing CAM with their providers included expectation of negative attitudes, lack of time or continuity, absence of reason to bring up the topic and simply because “Nobody asked”.

The most common sources of information about CAM among both CAM users and No CAM users were media (*n* = 214), family or friends (*n* = 154) and internet (*n* = 118). CAM therapists were less common as information sources (*n* = 35) and conventional care least common (*n* = 26). Other sources (*n* = 63) involved own experiences and interests, literature, lectures, courses, patient organizations and other therapists.

Most patients reported that they preferred receiving information on CAM during personal counselling with a skilled person (*n* = 354). Written information, e.g. webpages or patient brochures (*n* = 245) and lectures (*n* = 93) were other options, while relatively few (*n* = 42) wanted to chat online.

### Reasons for CAM use, perceived benefits, satisfaction and monthly costs

**Fig. 2:**
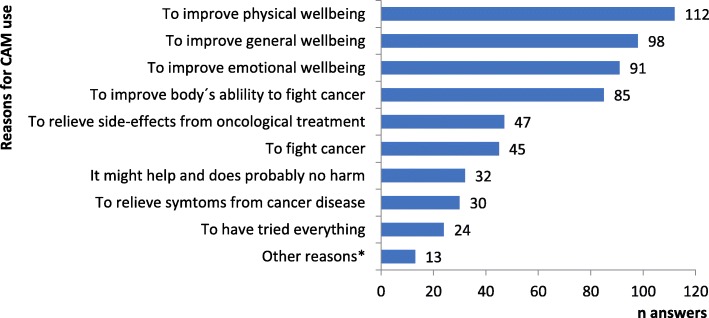
Reasons for CAM use among CAM using cancer patients. Quantity of answers per reason. ^*^ “other reasons” from free text option (see Table 2 Q9)

The most commonly reported reasons for CAM use were to improve physical and general well-being (Fig. 2). The majority of free-text responses were specifications of the pre-listed options in the multiple-choice question (see Table 2 Q9). For example, one woman specified: “To counteract strong hot flushes.” Only one response could be attributed to a reason for CAM use beyond the given options, i.e. because of declining conventional oncological treatment.

The explanations given not to use CAM were mainly satisfaction with received conventional cancer care, never having thought about CAM use or disbelieve in methods lacking scientific prove. Less frequent reported reasons were economy or discouraging advice from friends, family or cancer care.

**Fig. 3:**
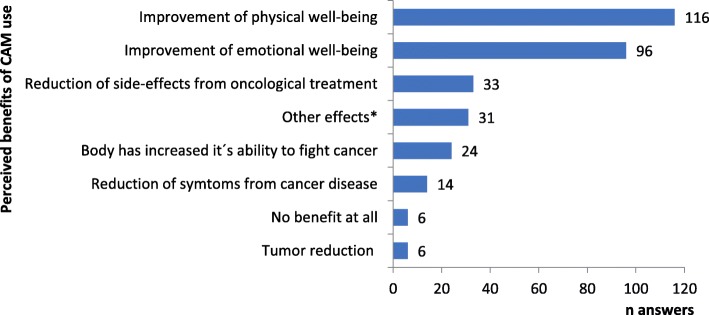
Perceived benefits of CAM. Quantity of answers per benefit. ^*^ “Other effects” from free text option (see Tableb 2 Q10)

Perceived benefits of CAM were mainly improved physical and emotional well-being (Fig. 3). The free-text responses related to perceived benefits did not diverge from the pre-listed options but were rather specifications of experiences in relation to CAM use, perceived effects or lack of effects, and thoughts regarding the difficulty of evaluating effect (see Table 2 Q10). For example, one woman stated: “Have experienced many side-effects from the antioestrogen treatment but my sexual life has not been affected, my mucous membranes are not dry. Because of the primrose oil? Not of any use: The acupuncture that even hurt sometimes.”.

Most participants (87%, *n*=113) who reported their grade of satisfaction (*n* = 130) were very (45%, *n* = 58) or quite (42%, *n* = 55) satisfied with their CAM use; 12% (*n* = 15) were a bit satisfied and 2% (*n* = 2) not satisfied at all. More than half of the patients reported spending ≤50 € monthly while 3% spent > 500 €. Over 90% of the patients reported that they considered their CAM therapies worth the cost (Table 5). In the free text responses related to expenses, several patients highlighted the value of CAM for life in general (see Table 2, Q13). One participant wrote: “I got a new life. Even if I would die tomorrow, it would have been worth it”.

**Table 5:** Monthly costs for CAM and reported value

Monthly costs € ^a^	CAM user % (n)	Worth the money % (n)	Not worth the money % (n)	Missing answer % (n)
0	20 (39)	–	–	–
1–50	34 (67)	88 (59)	0 (0)	12 (8)
51–100	16 (31)	94 (29)	0 (0)	6 (2)
101–500	10 (19)	74 (14)	11 (2)	16 (3)
501–1000	2 (3)	100 (3)	0 (0)	0 (0)
> 1000	1 (1)	100 (1)	0 (0)	0 (0)
Unknown	19 (38)	–	–	–
1- > 1000	61 (121)	88 (106)	2 (2)	11 (13)

^a^ Euro

### The role of cancer care in relation to CAM

Over two thirds of the CAM users and nearly half of No CAM users expressed that providers should be able to answer questions about CAM (Table 6) and that CAM therapies should be offered in the regular oncology settings (Table 7). The option to leave additional responses was used by 89 out of 198 CAM users: they addressed the importance of evidence (*n* = 42), competence and quality of CAM modalities that they thought should be included in oncological care, they suggested specific methods or indications, expressed the wish that providers should at least offer information on CAM modalities and specified a wish for treatment diversity.

**Table 6:** Should providers be able to inform?

Should cancer care provider be able to inform about CAM?	Patients % (n)	CAM user % (n)	No CAM user % (n)
Should be able to inform	53 (403)	67 (132)	49 (271)
Need not be able to inform	4 (30)	2 (4)	5 (26)
No opinion or missing answer	43 (322)	31 (62)	47 (260)
Total	100 (755)	100 (198)	100 (557)

**Table 7:** Should CAM be offered within conventional cancer care?

Should certain CAM modalities be offered?	Patients % (n)	CAM user % (n)	No CAM user % (n)
Should be offered	54 (411)	71 (141)	48 (270)
Should not be offered	23 (177)	15 (29)	27 (148)
Missing answer	22 (167)	14 (28)	25 (139)
Total	100 (755)	100 (198)	100 (557)

## Discussion

This study describes CAM use and related experiences among Swedish patients with solid tumors in both curative and palliative stage. One third (34%) of participants had used CAM at some point in their life and 26% *after* cancer diagnosis. This is in line with previous studies, e.g. a European study where CAM use was reported by 36% [3], without distinction between use before or after cancer diagnosis. The similarity to our findings is surprising as access to and advice on CAM modalities are generally low within cancer care in Sweden, due to Swedish regulations requiring health personnel to practice in accordance with “science *and* experiential knowledge” [27, 28]. Since patients in this study visited an oncological department, CAM seems to be mainly used complementary and not alternatively to conventional therapy. This also corresponds to participants´ free-text responses where they specify reasons for and benefits from CAM use.

In line with international [3, 29, 30] and other Scandinavian surveys [4, 9], we found statistically significant relationships between CAM use and being female, younger and higher educated. These results may suggest high health literacy among CAM users and a gender aspect of unmet needs in conventional care.

In agreement with previous results [3, 31] we found that patients used and combined a diversity of CAM modalities. Natural products and mind-body therapies were most popular. This concurrent and multimodal use implies an immense challenge for research and practice in regard to interactions [32], efficacy and educational needs among patients as well as providers in a pluralistic context [33].

In our study, 5.6% of CAM users reported mild and transient adverse effects (mostly gastrointestinal discomfort) from a CAM modality, which was mainly related to intake of natural products, as shown earlier [3, 9, 34]. Participants’ detailed specifications related to their experiences of CAM use as well as previous results on patients´ concerns about risks for interactions [35] indicate that many CAM users pay close attention to both positive and negative consequences of their CAM use. Thus, patients seem to be a potential and possibly underestimated resource for monitoring of adverse effects and effectiveness, as also acknowledged by the introduction of PROM/PREM in evaluating clinical trials. Therefore, an open and trustful dialogue between patients and providers is fundamental. However, in our study only 2% of No CAM users and 33% of CAM users had discussed CAM modalities with their provider, i.e. less than in recent Scandinavian literature [9, 10, 34]. The results from these previous studies indicate that around 50% of patients have a dialogue about CAM. Consequently, up to 67% of CAM use in our study may be unknown to providers representing both potential risks and undiscovered possibilities. Since CAM users were more likely to have discussed CAM compared to No CAM users, this dialogue was usually patient initiated, suggesting a threat to patient safety. For example, it has been shown previously that physicians who are perceived to be poorly informed or negative about CAM induce safety concerns in terms of e.g. potential undiscovered interactions as well as patient anxiety [35].

Two thirds (67%) of CAM users and 49% of No CAM users (Table 6) thought that providers should have enough knowledge to be able to answer questions on CAM; which has been shown earlier [11, 35]. However, conventional health care was the least common source of information about CAM modalities while media, family or friends and internet were most commonly used; as previously reported [31, 34] and highlighting the need for quality assured information. Our findings are not surprising since Swedish health care professionals still report lack of knowledge about CAM [11, 21, 36–38]. Better knowledge is known to increase dialogue [21–23].

The main reasons for CAM use were to improve well-being and to increase the body’s ability to fight cancer, i.e. not to fight cancer per se. Benefits were reported as being mostly physical and emotional. Both reasons for use and perceived benefits were often multifaceted, e.g. a single CAM modality was used and perceived efficacious both for improving emotional well-being and reducing adverse effects of conventional treatment. Notably, fighting cancer was rarely given as a reason for CAM use. These results correspond to earlier research indicating complex motives for CAM use [39–43], shifting motives over time [34, 44] and benefits not always related to initial reasons for use [3, 8, 30]. Also, in line with previous findings, [3] patients in this study reported high satisfaction with CAM (87%) and the majority of users considered CAM being worth the money (91% of CAM users). Moreover, 71% of CAM users and 48% of No CAM users considered that CAM modalities should be offered within conventional cancer care and participants stressed the importance of scientific evidence, competence and quality of CAM. While patients’ satisfaction with care - conventional or CAM - is multifaceted and clearly needs to be considered together with other aspects of evidence, as also patients argue in their responses, the high satisfaction rates among CAM users found here needs to be considered by decision makers in cancer care.

Strengths of this study include the cross-sectional design with three different data collection sites and the large sample size. These factors help ensure representativity for cancer patients with solid tumors in urban Sweden, although the results may not be fully transferable to more rural areas. The questionnaires were completed anonymously, and responses could not be linked to electronic health records or sociodemographic variables; thus, participants’ diagnoses and socio-demographics are exclusively self-reported. Self-selection bias potentially attracting CAM users to participate to a larger extent than No CAM users is however unlikely since gender proportions were similar in visits to the clinic and responses to the questionnaire. If CAM users had been more likely to respond to the questionnaire, there would be an overrepresentation of women among responders since female patients represented 80% of CAM users in our material.

Strengths with the questionnaire, although not formally validated, were that an earlier version had been previously used in a large European survey, and that space was given for additional free-text responses. The recruitment of participants at their follow-up visit at the oncology department might explain somewhat lower CAM use compared with previous studies. Terminally ill patients were not represented and in general, participants might have been reluctant to admit CAM use while waiting for conventional oncological care. The response rate of 58% could be seen as a limitation of the study and a higher response rate would have been desirable, however, it is in line with previously published CAM surveys [45].

## Conclusion

To conclude, the results here suggest that at least one fourth of Swedish cancer patients use CAM, are highly satisfied with this use, generally have reasonable expectations and are alert to the consequences. Therefore, our results point to an urgency of research on CAM and an informed professional practice to ensure patient safety and satisfaction. Cancer care professionals need to be able to discuss CAM based on the three principles of evidence-based medicine (patients’ values, professional experience, current research). Swedish cancer care needs a strategy for research and education about CAM to integrate CAM modalities with shown beneficial value for patients and to avoid possibly harmful CAM. The concept of integrative oncology [14] may provide a professional solution both regarding providers´ and patients’ needs.

## Additional file

Additional file 1: Questionnaire_eng_Wode et al_190128.pdf; English translation of used Swedish questionnaire. Description of data: Questionnaire containing 19 questions. (PDF 313 kb)

## Abbreviations

CAM: Complementary and Alternative Medicine
Q: Question
