# Supplementary material for: Cancer patients’ use of complementary and alternative medicine in Sweden: a cross-sectional study
Source: BMC Complement Altern Med. 2019 Mar 13;19:62. doi: 10.1186/s12906-019-2452-5 (PMC6417272; doi:10.1186/s12906-019-2452-5)
Supplement: Supplementary file 1 — Questionnaire_eng_Wode et al_190128.pdf; English translation of used Swedish questionnaire. Description of data: Questionnaire containing 19 questions. (PDF 313 kb) [file 12906_2019_2452_MOESM1_ESM.pdf]

If you have a scheduled appointment at the Department of oncology

We would appreciate if you could take a little time in the waiting room to answer some questions.

When receiving a cancer diagnosis, it is natural to think about one´s own contribution to improve the situation. We know that for many people, this involves trying other medications/dietary supplements or treatment methods than those recommended by doctors, so called complementary and alternative medicine.

Today, such methods are not regularly used within cancer care, since most have not been scientifically investigated.

We are therefore conducting this research project with the aim to describe the use of these methods among people with cancer in the Stockholm area. Data will be collected by regional experts for Integrative Cancer Care from Regional Cancer Center Stockholm-Gotland.

What does this mean for me?
Your participation in this project means that you consent to anonymously fill in your answers in the attached questionnaire containing 20 questions. It takes 5-10 minutes to fill in the questionnaire. Please return the questionnaire in the designated box in the waiting room after you have answered all the questions.

Your answers will be handled safely to ensure that no unauthorized persons can access the information. Your health care will in no way be affected by your decision to participate or not and your participation is entirely on a voluntary basis.

Research has ethical rules; information that may be associated with a certain individual will not be revealed. Cases that may be discussed for research purposes will always exclude any given names of patients and personal data. The research is evaluated by the Ethical board. Results will be published in scientific journals.

Thank you for your help!

Responsible researchers are:

Kathrin Wode, M.D. Oncologist &
Johanna Hök, lic. Pharmacist, PhD.

Contact: kathrin.wode@karolinska.se, 08-616 69 72

1. **Age:** ____

2. **Are you**

☐	Man
☐	Woman

3. **Where in the body did your cancer start?** ______________________________

4. **What is the highest educational level you have completed?**

☐	Elementary school
☐	High school
☐	College/university
☐	Post-graduate education

5. **Have you <u>at any point in life</u> used any form of so called complementary and alternative methods?**

☐ Yes → *Continue with question 7.*

☐ No → *Continue with question 6 and then question 15-19.*

6. **Please check the main reason(s) for not using any complementary and alternative methods:**
Check all that apply.

☐	Never thought about using complementary and alternative methods
☐	I do not believe in methods lacking scientific proof
☐	I am happy with the care and treatment I receive at the department of oncology
☐	I cannot afford complementary and/or alternative methods
☐	I´ve thought about using complementary and alternative methods but was discouraged family/friends/health professionals and decided therefore not to do so
☐	Other reason: ______________________________

7. Have you used any of the following complementary and alternative methods before or after your cancer diagnosis? What of these methods are you currently using?
Check all that apply.

	**Used before cancer diagnosis**	**Used after cancer diagnosis**	**Using now**
Ayurveda	☐	☐	☐
Acupuncture	☐	☐	☐
Spiritual guidance, healing	☐	☐	☐
Anthroposofic medicine	☐	☐	☐
Aroma therapy	☐	☐	☐
Relaxation	☐	☐	☐
Prayer	☐	☐	☐
Energy medicine	☐	☐	☐
Homeopathy	☐	☐	☐
Hypnosis	☐	☐	☐
Art therapy	☐	☐	☐
Changes in diet beyond those recommended at the department of oncology	☐	☐	☐
Laser therapy	☐	☐	☐
Massage	☐	☐	☐
Mindfulness	☐	☐	☐
Meditation	☐	☐	☐
Mistletoe injections	☐	☐	☐
Naprapathy, Chiropractic treatment	☐	☐	☐
Natural products incl. Echinacea, omega-3, arctic root, garlic. *Please specify:*	☐	☐	☐
______________________	☐	☐	☐
______________________	☐	☐	☐
______________________	☐	☐	☐
______________________	☐	☐	☐
______________________	☐	☐	☐
Rosen therapy	☐	☐	☐
Shiatsu	☐	☐	☐
Support groups	☐	☐	☐
Tai Qi/Qi gong	☐	☐	☐

Cont. question 7.	Used before cancer diagnosis	Used after cancer diagnosis	Using now
Traditional Chinese Medicine	☐	☐	☐
Vitamins and minerals *Please specify:*	☐	☐	☐
	☐	☐	☐
______________________	☐	☐	☐
______________________	☐	☐	☐
______________________	☐	☐	☐
______________________	☐	☐	☐
Yoga	☐	☐	☐
Reflexology	☐	☐	☐
**Others, please specify:**	☐	☐	☐
______________________	☐	☐	☐
______________________	☐	☐	☐

If you have not used any of the methods above **after** your cancer diagnosis →*Continue with question 15.*

8. If you began using complementary and/or alternative therapies after you knew you had cancer, when did you begin using these therapies?

____
____
____

9. Please give the reason why you are using or have used complementary and alternative methods.

Check all that apply.

☐	To fight cancer
☐	To improve the body´s ability to fight cancer
☐	To improve physical well-being
☐	To improve emotional well-being
☐	To improve general well-being
☐	To relieve symptoms from cancer disease
☐	To relieve side-effects from oncological treatment
☐	” It might help and does probably no harm.”
☐	To have tried everything
☐	Other:

10. What benefit have you experienced form the used complementary and alternative methods?

Check alt that apply.

☐	Direct decrease of tumor as a result from using complementary and alternative methods
☐	Increase in my body´s ability to fight cancer
☐	Improvement of physical well-being
☐	Improvement of emotional well-being
☐	Reduction of symptoms from cancer disease
☐	Reduction of side-effects from oncological treatment
☐	Other:
☐	No benefit at all

11. Have you experienced any ill effects or side-effects, which you attribute to the complementary and alternative therapy?

☐ Yes → *Specify **what** and in relation to what **method** below.*

☐ No

Method	Ill effect or side-effect

12. How much do you currently spend on complementary and alternative methods (incl. products and treatments) in one month? (Please check)

☐	0 SEK
☐	500 SEK or less
☐	501-1000 SEK
☐	1001-5000 SEK
☐	5001-10 000 SEK
☐	10 000 SEK or more

13. Do you think the complementary and alternative methods you have used were worth the money?

☐ Yes

☐ No

Please comment:

____

____

____

14. How satisfied are you with the complementary and alternative method(s) you have used since your cancer diagnosis?

☐ Not satisfied at all ☐ A bit satisfied ☐ Quite satisfied ☐ Very satisfied

Please comment:

____

____

____

15. Have you discussed use of complementary and alternative methods with your doctor or nurse at the department of oncology?

☐ Yes →*Continue with question 15a-15b.*

☐ No → *Continue with question 15c.*

15a. If yes, what was his/her answer?

Specify with whom (doctor or nurse) you have discussed this use.

____

____

15b. If yes, were you satisfied with the answer?

☐ Yes

☐ No

Please comment:
____
____
____

15c. If no, please state the reason
____
____

16. What was your source of information about complementary and alternative methods?
Check all that apply.

☐	No one, never heard of it until today
☐	The media (TV, magazines, newspapers)
☐	Internet
☐	Family and friends
☐	Practitioners of complementary and alternative therapies
☐	From primary care/department of oncology/home care
☐	Other: ____

17. In your opinion, should health care providers at the oncology department be able to answer questions related to complementary and alternative methods?

☐ Yes

☐ No

☐ No opinion

18. How would you prefer to receive information about complementary and alternative methods from the oncology department?

☐	Written information (webpage or brochure)
☐	E-mail/chat forum with possibility to ask questions
☐	Personal meeting with a competent person
☐	Lecture
☐	Other: ____

19. In your opinion should certain complementary and alternative methods be offered within oncological care?

☐ Yes

☐ No

Please comment:

____

____

Thank you for your participation!
